# Climate Actions, Persistent Pollutants, and Human Health: A Call for Integrated Assessments

**DOI:** 10.1021/acs.est.4c07707

**Published:** 2024-08-28

**Authors:** Shan Niu, Minghao Qiu, Li Li, Chenfei Qu, Da Zhang

**Affiliations:** † Advanced Interdisciplinary Institute of Environment and Ecology, Beijing Normal University, Zhuhai 519085, China; ‡ School of Marine and Atmospheric Sciences, Stony Brook University, Stony Brook, New York 11794, United States; # Program in Public Health, Department of Family, Population and Preventive Medicine, Stony Brook University, Stony Brook, New York 11794, United States; § School of Public Health, University of Nevada, Reno, Nevada 89557, United States; ∥ Institute of Energy, Environment, and Economy, Tsinghua University, Beijing 100190, China

**Keywords:** climate mitigation strategies, environmental contaminants, persistent organic pollutants, health risk assessment, sustainable policies

In response to climate change, more than 150 countries have established targets for reducing greenhouse gas emissions. These decarbonization objectives and proposed climate policies hold promise for mitigating the release of co-emitted air pollutants from fossil fuel combustion, thus offering benefits for human health through improving air quality.^1^ One challenge in assessing the impacts of climate policies on air quality and human health is the limited focus on certain pollutants. Studies have predominantly focused on the effects of policy on particulate matter, ozone, and their precursors. Limited attention has been paid to organic pollutants that could potentially be carcinogenic, mutagenic, and toxic, such as persistent organic pollutants (POPs) and other emerging contaminants, whose sources are closely linked to industrial processes and fossil fuel combustion. The objective of this Viewpoint is to provide perspectives on developing and selecting integrated climate mitigation measures for human health, by expanding the range of environmental pollutants assessed.

POPs and other emerging contaminants have been widely detected in the air and a multitude of environmental and biological matrices.^2^ Among these, certain POPs, such as per- and polyfluoroalkyl substances (PFAS), are intentionally synthesized and utilized across industrial and consumer sectors, including automotive, construction, and electroplate industries, as well as in everyday consumer products like carpets, furniture, and outdoor equipment. Some PFAS are persistent in the environment, bioaccumulative in human tissues, and associated with a wide range of health issues, including kidney cancer, liver dysfunction, immune system disruption, and reproductive and developmental toxicity.^3^ Estimates suggest that the economic burden attributed to PFAS-related diseases in the United States ranged from $5.52 billion to $62.6 billion.^3^ In addition to intentional production, certain POPs are unintentionally formed from thermal processes and combustion of fossil fuels. Examples include polychlorinated dibenzo-*p*-dioxins and dibenzofurans (PCDD/Fs), polychlorinated naphthalenes (PCNs), polychlorinated biphenyls (PCBs), and polycyclic aromatic hydrocarbons (PAHs) (which are classified as POPs under the United Nations Economic Commission for Europe). The production and use of technical mixtures were once the major sources of PCBs; however, wood fires, industrial activities, biomass and fossil fuel combustion, and other combustion processes have become significant sources of unintentionally produced PCBs.^4^ PCDD/Fs, PCNs, PCBs, and PAHs pose various health risks, being identified as hepatotoxic, neurotoxic, carcinogenic, endocrine-disrupting, and developmental toxicants.^5^ Emission sources of these unintentionally generated POPs include power plants, heavy and light industries, transportation, and residential activities. These emission sources and emission factors (the amount of pollutants produced per unit of energy) might change alongside the evolution of the industrial landscape and technological advancements.

The reduction in the level of fossil fuel combustion, depending on specific approaches, may mitigate human exposure risks related to combustion-related POPs in the proposed climate actions. However, different mitigation measures can lead to distinct implications for the emissions of POPs. For example, substituting coal with biomass may increase the emissions of unintentionally produced POPs. In addition, biomass or carbon capture and storage, while effectively reducing CO_2_ emission, will not decrease the emission of POPs and other contaminants. In contrast to short-lived climate pollutants, these chemicals can persist in the air, settle into soil and water, and build up in human tissues, leading to prolonged exposure and long-term adverse health effects.

The transition from fossil fuel-based energy to renewable and clean energy may also result in the emission of POPs and other contaminants. The development and production of clean energy sources, such as green hydrogen, wind, and solar energy, involve the use of PFAS. PFAS are widely used in products such as fuel cells, wind turbines, and photovoltaic solar panels, given their excellent insulation properties and resistance to physical, thermal, and chemical challenges. In addition, PFAS are used in rechargeable batteries to ensure optimal performance. As these batteries are increasingly used in electric vehicles, facilitating the shift toward electric transportation, the widespread use of PFAS-containing rechargeable batteries and clean energy technologies, along with their recycling processes, is likely to increase the emissions of PFAS into the environment, potentially posing risks to human health. While climate policies can help improve human health directly through avoiding disastrous climate change, the channels we highlight here suggest that policymakers should take POPs into account when evaluating and designing climate policies for a more comprehensive assessment of the effects of human health.

We advocate for a collaborative approach that bridges multiple disciplines, fostering interfaces between different policies (e.g., climate policy and POPs policy), as well as policy making and scientific research. An integrated framework is essential for addressing the following three key aspects: (1) emission inventories of POPs and other emerging contaminants under different climate actions, (2) quantitative health risk and cost assessments associated with these pollutants, and (3) development of effective climate mitigation strategies with integrative health risks considered. Only through a comprehensive assessment framework, which considers the effects of policy on both the climate forcings and co-emitted pollutants, can we effectively navigate the complex interplay among climate actions, environmental pollutants, and human health, thereby safeguarding both environmental and public health interests.
